# Busulfan administration produces toxic effects on epididymal morphology and inhibits the expression of ZO-1 and vimentin in the mouse epididymis

**DOI:** 10.1042/BSR20171059

**Published:** 2017-12-12

**Authors:** Fang Fang, Ke Ni, Yiting Cai, Qian Zhao, Jin Shang, Xiaoke Zhang, Shiliang Shen, Chengliang Xiong

**Affiliations:** 1 Family Planning Research Institute, Tongji Medical College, Huazhong University of Science and Technology, Wuhan, China; 2 Department of Anesthesiology, Tongji Hospital, Tongji Medical College, Huazhong University of Science and Technology, Wuhan, China; 3 Department of Obstetrics and Gynecology, Center of Reproductive Medicine, Second Affiliated Hospital, Zhejiang University School of Medicine, Hangzhou, China; 4 Department of Pathology, Zhong Shen Bioscience Inc., Wuhan, China; 5 Center of Reproductive Medicine, Wuhan Tongji Reproductive Medicine Hospital, Wuhan, China

**Keywords:** Busulfan, Blood-epididymis barrier, Epididymis, Vimentin, ZO-1

## Abstract

Busulfan is an alkane sulphonate currently used as an anticancer drug and to prepare azoospermic animal models, because it selectively destroys differentiated spermatogonia in the testes. However, few studies have focussed on the exact effects of busulfan treatment on the epididymis currently. The present study assessed the effect of busulfan on epididymal morphology and the blood–epididymis barrier in mice. We treated mice with a single injection of busulfan and detected the effect at different time points. We showed that busulfan was toxic to the morphological structure and function of the epididymis. Furthermore, busulfan treatment down-regulated the epididymal expression of vimentin and zonula occludens-1 (ZO-1) at the mRNA and protein levels. In addition, there was an increase in total androgen receptor (AR) levels, whereas the estrogen receptor-α (ER-α) levels were reduced, both in the caput and cauda regions after busulfan treatment, which may be secondary to the testicular damage. In conclusion, our study describes the effects of busulfan administration on the mouse epididymis and also provides a potential understanding of male infertility arising from chemotherapy-related defects in the epididymis.

## Introduction

The epididymis plays an important role in post-testicular sperm maturation including the acquisition of forward motility and fertilizing ability [1,2]. When transiting through the lumen of the epididymis, sperm undergo maturation by interacting with proteins synthesized and secreted by the epididymal epithelium [3]. In addition, the epididymis also functions in sperm transport and concentration, immunoprotection of sperm, and serves as a sperm reservoir [3,4]. Generally, the epididymis is divided into four main anatomical regions: the initial segment (only present in rodents), the caput, the corpus and the cauda, with each epididymal region performing separate functions essential to the different steps of sperm maturation. In support of this view, early studies demonstrated that the caput and corpus provide microenvironments for sperm maturation, and that the cauda region primarily serves as a storage site for functional spermatozoa [5,6].

The adult epididymal epithelium consists of different cell types, including principal, clear, basal, halo, narrow, and apical cells, which form a monolayer surrounding the lumen [3]. Although these cell types within the epididymal epithelium have individual functions, they also communicate with each other to maintain sperm maturation and storage via different cell junctions [7]. The principal cells form tight junctions between adjacent cells to create the blood–epididymis barrier, which is necessary for the stable and specific microenvironment within the epididymal lumen. In the epididymis, epithelial cell–cell interactions are closely associated with cadherin-mediated cell adhesion. Occludin, cadherin, and tight junctional protein 1 (also known as zonula occludens-1 (ZO-1)) are implicated in the formation of epididymal tight and adherens junctional complex [8]. Gap junctions formed by a family of integral proteins known as connexins, are also present between adjacent principal cells [3]. Thus, epithelial cell cross-talk by cell junctions in the epididymis is critical for the luminal microenvironment, which is responsible for sperm maturation.

Busulfan is a chemotherapeutic and cytostatic agent that is widely used to remove endogenous germ cells from the testes of animal models [9,10]. As a common chemotherapeutic agent for hematological diseases, busulfan has been reported to cause infertility in oncology patients [11,12], and germinal epithelial damage is a recognized consequence of busulfan treatment in mice [13]. Unlike other chemicals that destroy differentiated spermatogonia, busulfan eliminates spermatogonial stem cells preferentially [14]. There is growing evidence that busulfan treatment causes deleterious effects on cell junctions and the cytoskeleton. Intercellular adhesion molecule-1 (ICAM-1) is involved in impaired spermatogenesis after busulfan treatment in mice [15]. The expression of P-cadherin decreases when spermatocytes disappear after busulfan treatment [16]. Cyclophosphamide, another alkylating agent, induces destructive changes in the structure of the blood–testis barrier by interference with the cytoskeleton of Sertoli cells [17].

The epididymis is highly androgen-dependent and is responsible for maintaining epididymal structure and functions. In the epididymis, testosterone bound to androgen-binding protein (ABP) is taken up by the principal cells of the initial segment and caput epididymidis through a receptor-mediated process [18]. Treatment with busulfan has a deleterious effect on testis structure and functions, which might result in the loss of androgens entering the epididymis. Few studies have focussed on the androgen actions mediated by the androgen receptor (AR) in the epididymis after busulfan treatment.

Currently, there is a critical lack of information as to the exact effects of busulfan treatment on the epididymis, mainly because most studies have focussed on seminiferous epithelial damage in the testis. A precise understanding of the influence of busulfan on the epididymis might have significant implications for male reproductive biology in general as well as a broad range of male infertility arising from chemotherapy-related defects in the epididymis. Therefore, the aim of the present study was to assess the effect of busulfan administration on the mouse epididymis.

## Materials and methods

### Animals and treatments

Adult male ICR mice (6 weeks old) were purchased from the Center for Disease Control and Prevention, Hubei Province, China and kept in an isolated environment under controlled light conditions (12-h light/12-h dark cycle), temperature (25 ± 3°C) and humidity (50 ± 5%) with free access to food and water. Animals were weighed and then treated with a single i.p. injection of busulfan (Sigma, St. Louis, MO, U.S.A.) at a dose of 40 mg/kg. Busulfan was dissolved in DMSO (Sigma) to a final concentration of 10 mg/ml. Animals in control groups were injected with the same volume of DMSO or saline. Mice in different groups were killed by cervical dislocation at the end of weeks 1, 2, 3, and 4 of treatment. Following cervical dislocation, the testis and epididymis were weighed and collected for subsequent experiments. The present study was approved by the Institutional Animal Care and Use Committee of Huazhong University of Science and Technology.

### Histological and morphometrical analyses

Testicular and epididymal tissues were fixed in Bouin’s solution and dehydrated in alcohol before embedding in paraffin. Sections of 5-μm thickness were stained with Hematoxylin and Eosin. At least three sections per testis or epididymis in each group were observed in an optical microscope (Olympus, Japan). Metrical software (cellSens, Olympus, Japan) was used to measure the diameters and epithelial thickness of epididymal tubules in both the caput and cauda segment by optical microscopy. The diameters of epididymal tubules were determined by the average value of the longitudinal and transverse cross-sections. An estimate of each parameter was performed by examining 15 fields in three histological sections from each epididymis.

### Quantitative real-time PCR

RT-PCR was performed to detect the expression of cell junction and cytoskeleton-related genes in the epididymis after busulfan treatment. Briefly, TRIzol reagent (Invitrogen, Waltham, MA, U.S.A.) was used to extract total RNA from epididymal tissues according to the manufacturer’s instructions. RNAs were then reverse transcribed into cDNA with the RevertAid First Strand cDNA Synthesis Kit (Thermo Scientific, Waltham, MA, U.S.A.). Quantitative real-time PCR was performed with SYBR Premix Ex Taq II (TaKaRa Biotech Co., Ltd., Dalian, China) using the LightCycler® 96 SW 1.1 real-time PCR detection system (Roche, Mannheim, Germany). The primers used to detect the tissue-specific genes in the present study are shown in Supplementary Table S1 and β-actin was used as an internal control.

### Immunofluorescent staining

After deparaffinization and hydration in an ethanol gradient, the sections were boiled in EDTA buffer for 8–10 min in a microwave oven for heat-induced antigen retrieval. Next, the sections were treated with 3% (v/v) H_2_O_2_ in distilled water for 20 min to eliminate endogenous peroxidase. After being blocked in PBS with 5% (v/v) goat serum for 1 h at room temperature, the sections were exposed to primary antibodies at 4°C overnight. Primary antibodies were rabbit polyclonal anti-vimentin (1:200, Abcam, Cambridge, MA, U.S.A.) and rabbit polyclonal anti-ZO-1 (1:50, Santa Cruz, Dallas, TX, U.S.A.). Secondary antibody was goat anti-rabbit IgG (Cy3-conjugated, 1:200, Abcam), which was incubated with the sections for 1 h at room temperature. The cell nuclei were stained with DAPI (Vector Laboratories, Burlingame, CA, U.S.A.) and sections were assessed in a fluorescence microscope (Olympus).

### Immunohistochemistry

After the same process described above, the sections were incubated with rabbit polyclonal anti-AR antibody (1:80, Abcam) and rabbit polyclonal anti-estrogen receptor-α (ER-α) (1:100, Abcam) at 4°C overnight. After being washed in PBS three times, the sections were subsequently incubated with a secondary biotinylated anti-rabbit antibody (1:200, Aspen, Wuhan, China). The immunohistochemical staining was visualized with a DAB detection kit (DAKO, Glostrup, Denmark) and the sections were counterstained with Hematoxylin, dehydrated, cleared, and mounted. Sections incubated without primary antibody were also included in each staining experiment as a negative control to detect nonspecific binding of the secondary antibodies. Sections were assessed using an optical microscope (Olympus). IPP 6.0 software was used to measure the integral optical density (IOD) for quantitative analysis.

### Western blot analyses

Total proteins were extracted from epididymal tissues using Tissue Protein Extraction Reagent (Thermo Scientific) containing 1 mM of PMSF (Roche). The protein concentrations were determined by BCA Protein Assay Kit (Thermo Scientific). Equal amounts of protein samples were separated by SDS/PAGE (10% (w/v) gel) and transferred on to PVDF membranes (Millipore, Billerica, MA, U.S.A.). The transferred proteins were incubated with polyclonal rabbit anti-ZO-1 (1:500, Santa Cruz Biotechnology), polyclonal rabbit anti-vimentin (1:2000, CST, Danvers, MA, U.S.A.), and polyclonal rabbit anti-GAPDH (1:10000, Abcam) primary antibodies at 4°C overnight, followed by incubation with the horseradish peroxidase conjugated anti-rabbit (1:10000, KPL, Milford, MA, U.S.A.) secondary antibody for 1 h at 37°C. The reaction was developed with the ECL kit (Pierce Chemical, Dallas, TX, U.S.A.) and photographed.

### Terminal deoxynucleotidyl transferase dUTP nick-end labeling staining

To investigate the cell apoptosis, an *in situ* cell death detection POD Kit (Roche) was used for the terminal deoxynucleotidyl transferase dUTP nick-end labeling (TUNEL) technique and the sections were stained according to the manufacturer’s instructions for paraffin-embedded tissues. We visualized the POD retained in the immune complex with a DAB detection kit (DAKO). Sections were assessed using an optical microscope (Olympus).

### Statistical analyses

The results are expressed as the mean ± S.D. of at least three independent experiments. One-way ANOVA was applied for multiple comparisons. In addition, an independent-sample Student’s *t*test was performed to compare normally distributed sample groups. All statistical analyses were performed using SPSS 18.0 software (SPSS Inc., Chicago, U.S.A.), and a value of *P*<0.05 was considered significant.

## Results

### Assessment of mouse body, testicular, and epididymal weights at different time points following busulfan treatment

As presented in Table 1, testicular weights in the busulfan-treated group were decreased at all time points compared with control groups. There was also an obvious decline in epididymal weights 3 weeks after busulfan treatment. Accordingly, testicular and epididymal weights both reached the lowest level at the end of the 4th week. However, there was no difference between the body weights of the busulfan-treated and control groups (Table 1).

**Table 1:** Body, testicular, and epididymal weights in the control and busulfan-treated groups during the 4 weeks

Groups	Body weights (g)				Testicular weights (mg)				Epididymis weights (mg)			
	Week 1	Week 2	Week 3	Week 4	Week 1	Week 2	Week 3	Week 4	Week 1	Week 2	Week 3	Week 4
Bulsufan	30.03 ± 2.15	29.47 ± 4.94	32.87 ± 10.82	30.98 ± 6.39	79.57 ± 7.73*	94.63 ± 12.79*	70.22 ± 4.29*	38.57 ± 5.68*	24.03 ± 1.46	26.37 ± 3.47	30.40 ± 3.80*	21.73 ± 3.00*
DMSO	31.40 ± 2.72	31.67 ± 4.67	36.43 ± 7.15	40.80 ± 3.05	106.30 ± 9.34	114.93 ± 30.83	129.05 ± 24.00	121.23 ± 24.00	27.67 ± 3.68	30.67 ± 3.59	39.03 ± 3.86	38.88 ± 7.92
Saline	32.17 ± 2.57	35.63 ± 2.47	36.50 ± 8.41	31.54 ± 5.76	112.87 ± 7.40	119.67 ± 13.79	141.08 ± 18.43	119.87 ± 23.35	24.60 ± 0.94	30.90 ± 2.63	39.25 ± 2.95	33.36 ± 10.28

The replication of experiments was at least thrice for all the groups; *P<0.05, as compared with saline and DMSO groups at respective weeks.

### Analyses of morphological changes in the mouse testis and epididymis following busulfan treatment

**Figure 1:**
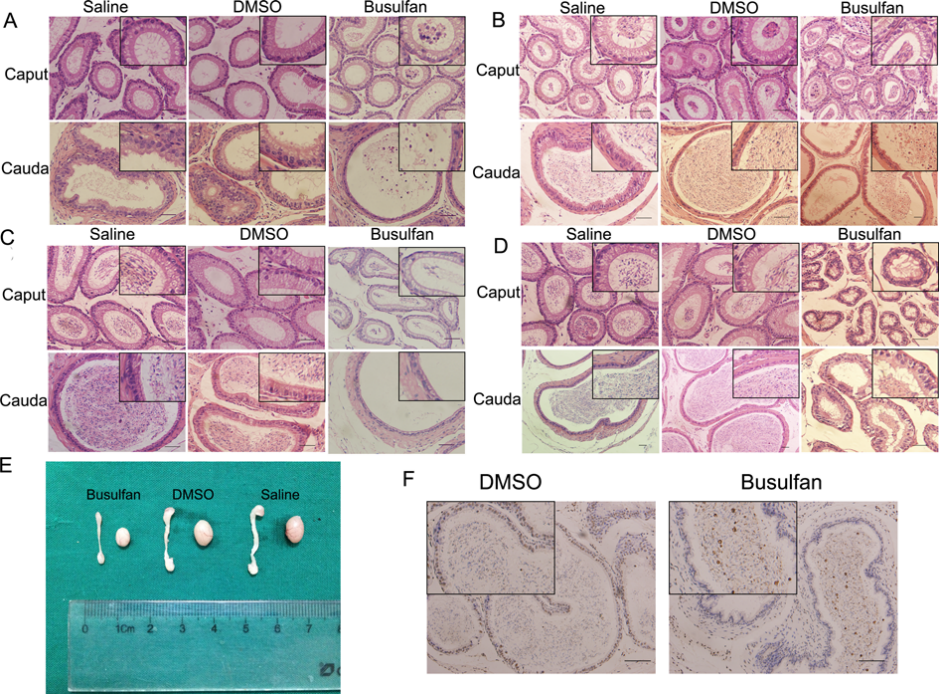
Histological examination of the caput and cauda epididymidis in mice following busulfan and control treatment The 1st (**A**), 2nd (**B**), 3rd (**C**), and 4th (**D**) week after treatment. (**E**) Photograph of the epididymis and testis at the end of the 4th week. (**F**) TUNEL staining for the cauda epididymidis at the end of the 2nd week. Arrows show round apoptotic cell bodies in the lumen of the cauda epididymidis after busulfan treatment. Bar: 50 μm.

Histological evaluations revealed that a single injection of 40 mg/kg busulfan in mice completely destroyed the germinal epithelium of the seminiferous tubules and decreased the sperm count in the epididymal tubules at the end of the 4th week (Supplementary Figure S1D and Figure 1D). There were no obvious morphological differences between the busulfan group and control groups after 1 week of busulfan treatment (Supplementary Figure S1A). At the end of the 2nd week, the germinal cells in the seminiferous tubules began to show vacuolation with disorganized germinal epithelium compared with the control groups which had normal testicular architecture and germinal cell arrangement (Supplementary Figure S1B). During the next week, the seminiferous tubules appeared to be compressed with noncohesive germinal cells separated from the basement membrane and the number of germinal cells markedly decreased. Leydig cells also appeared vacuolated in the busulfan-treated group (Supplementary Figure S1C). After 4 weeks, most of the germinal cells had been lost in the highly compressed seminiferous tubules with a thin layer of basal compartment. Most abnormal seminiferous tubules had only a single layer of Sertoli cells attached to the tubular basal lamina, the intertubular space was increased because of the atrophic seminiferous tubules, and the Leydig cells were in a state of compression at the end of the 4th week (Supplementary Figure S1D).

In the epididymis, there was an obvious decrease in the epididymal epithelial cell height in the caput; moreover, the cauda epididymidal sperm density in the busulfan group was dramatically decreased and there were large numbers of exfoliated germ cells and round bodies in the lumen but not in the control group by 3 weeks (Figure 1A–C). At the end of the 4th week, atrophic alterations in the epididymis including epithelial cell disorganization, cytoplasmic atrophy, vacuolation, and the consequent reduction in epithelial height appeared both in the proximal caput and cauda regions (Figure 1D). The result of TUNEL staining for the cauda segment of the 2nd week showed many round apoptotic cell bodies not found in the controls (Figure 1F). We observed that DMSO, the busulfan solvent, had no negative impact on the structure and morphology of the testis and epididymis, which were similar to those in the saline group. Images of the epididymis dissected from the testes confirmed that the size of the epididymis and testis was decreased in the busulfan group at the end of the 4th week (Figure 1E).

### Analyses of morphometrical changes in mouse epididymis following busulfan treatment

Compared with control groups, the diameters of the epididymal tubules were decreased in the busulfan-treated group both in the caput and cauda segments (*P*<0.05, from the 2nd and 3rd weeks, respectively) (Table 2). Accordingly, the thickness of the epididymal epithelium was obviously lower than that of the control groups as soon as 1 week after busulfan administration both in the caput and cauda segments (*P*<0.05) (Table 3).

**Table 2:** Epididymal tubule diameters of the mouse epididymis in the control and busulfan-treated groups during the 4 weeks

Groups	Caput (µm)				Cauda (µm)			
	Week 1	Week 2	Week 3	Week 4	Week 1	Week 2	Week 3	Week 4
Bulsufan	70.33 ± 5.30	73.06 ± 7.45	71.44 ± 12.29*	57.43 ± 10.21*	294.91 ± 59.20	259.28 ± 58.65*	273.09 ± 37.40*	219.34 ± 79.19*
DMSO	81.87 ± 14.09	106.51 ± 36.12	138.14 ± 26.47	122.49 ± 40.49	248.10 ± 116.16	391.53 ± 161.78	354.07 ± 50.80	462.11 ± 56.49
Saline	77.02 ± 11.28	89.81 ± 13.28	127.58 ± 32.43	121.33 ± 2.58	210.00 ± 31.98	368.92 ± 115.30	451.54 ± 139.21	500.54 ± 66.17

The replication of experiments was at least thrice for all the groups; *P<0.05, as compared with saline and DMSO groups at respective weeks.

**Table 3:** Epididymal epithelial thickness of the mouse epididymis in the control and busulfan-treated groups during the 4 weeks

Groups	Caput (µm)				Cauda (µm)			
	Week 1	Week 2	Week 3	Week 4	Week 1	Week 2	Week 3	Week 4
Bulsufan	30.75 ± 8.76*	28.25 ± 3.82*	21.67 ± 4.43*	20.99 ± 2.99*	25.14 ± 5.54*	26.75 ± 4.74*	24.78 ± 3.34*	22.60 ± 5.00*
DMSO	48.27 ± 7.27	44.44 ± 11.35	49.82 ± 5.83	52.78 ± 7.00	59.90 ± 12.45	53.74 ± 9.60	36.71 ± 12.09	30.58 ± 6.49
Saline	40.05 ± 6.58	50.22 ± 5.28	50.52 ± 6.68	46.66 ± 4.13	58.40 ± 14.22	55.45 ± 11.93	37.55 ± 11.14	34.43 ± 4.57

The replication of experiments was at least thrice for all the groups; *P<0.05, as compared with saline and DMSO groups at respective weeks.

### Cell junction- and cytoskeleton-related gene expression in the busulfan- and DMSO-treated groups

**Figure 2:**
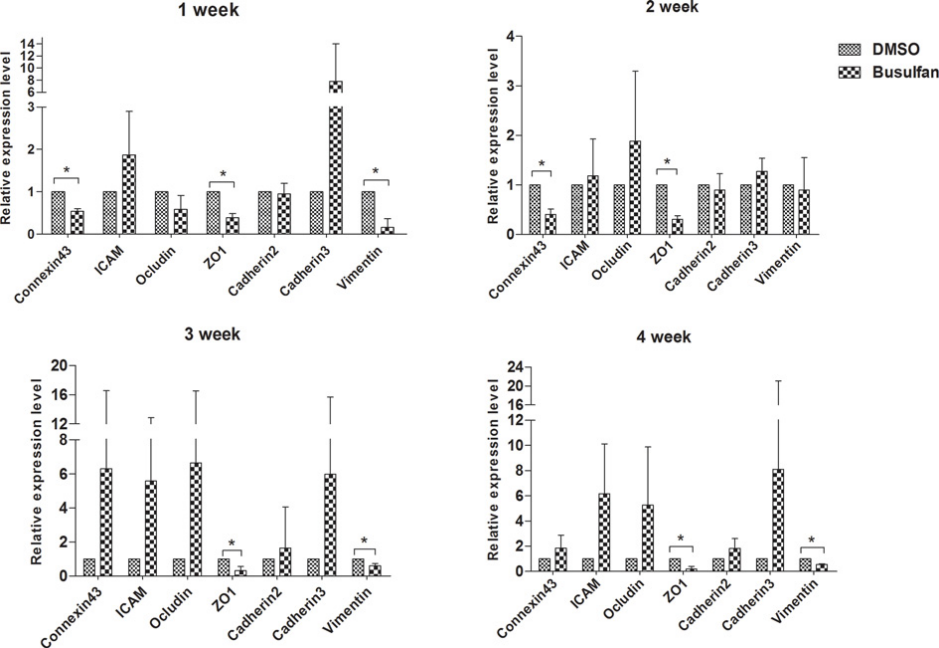
Relative expression of cell junction- and cytoskeleton-related genes in the mouse epididymis after busulfan treatment *n*=3, error bars denote ± S.D. *Significant difference (*P*<0.05), busulfan group compared with DMSO group.

We examined the cell junction- and cytoskeleton-related genes’ expression dynamics during busulfan treatment by qPCR (Figure 2). Primers used for RT-PCR are listed in Table 4. At the end of the 1st and 2nd weeks, the busulfan-treated epididymis exhibited lower levels of connexin43 than the DMSO group (*P*<0.05), whereas no significant differences were found between the busulfan and DMSO groups during the following 2 weeks (*P*>0.05). Additionally, the busulfan-treated epididymis exhibited down-regulated levels of ZO-1 and vimentin as early as the 1st week and remained at a lower level than in the DMSO group until the end of the 4th week (*P*<0.05), except for the vimentin level at the end of the 2nd week (*P*>0.05). The expression of ICAM, occludin, cadherin2, and cadherin3 showed no statistical difference between the two groups (all *P*>0.05).

**Table 4:** Primers used for RT-PCR

Genes	Forward primer	Reverse primer
*Connexin43*	5′-GGGAAAGGCGTGAGGGAAGT-3′	5′-GAGGCTGAAGTCTTTGGAAAAGG-3′
*ICAM-1*	5′-GGAAGGGAGCCAAGTAACTGTGAAG-3′	5′-GAGCGGCAGAGCAAAAGAAGC-3′
*Occludin*	5′-CTATGGGACAGGGCTCTTTGGA-3′	5′-AGGAAGCGATGAAGCAGAAGGC-3′
*ZO-1*	5′-GAGTGGACTATCAAGTGAGCCTAA-3′	5′-ATCCAAGTTGCTCGTCAATCTAA-3′
*Cadherin2*	5′-CCCAAGTCCAACATTTCCATCC-3′	5′-CTTTATCCCGCCGTTTCATCC-3′
*Cadherin3*	5′-GCTGCCAACACTGACCCTACT-3′	5′-CATCCTCACCGCCACCATACAT-3′
*Vimentin*	5′-AGAGCACCCTGCAGTCATTCAGA-3′	5′-CACTTTACGTTCAAGGTCAAGAC-3′
*β-actin*	5′-CTACCTCATGAAGATCCTCACCGA-3′	5′-TTCTCCTTAATGTCACGCACGATT-3′

### Immunocytochemical localization of ZO-1 and vimentin in the mouse epididymis and expression changes at the protein level after busulfan treatment

**Figure 3:**
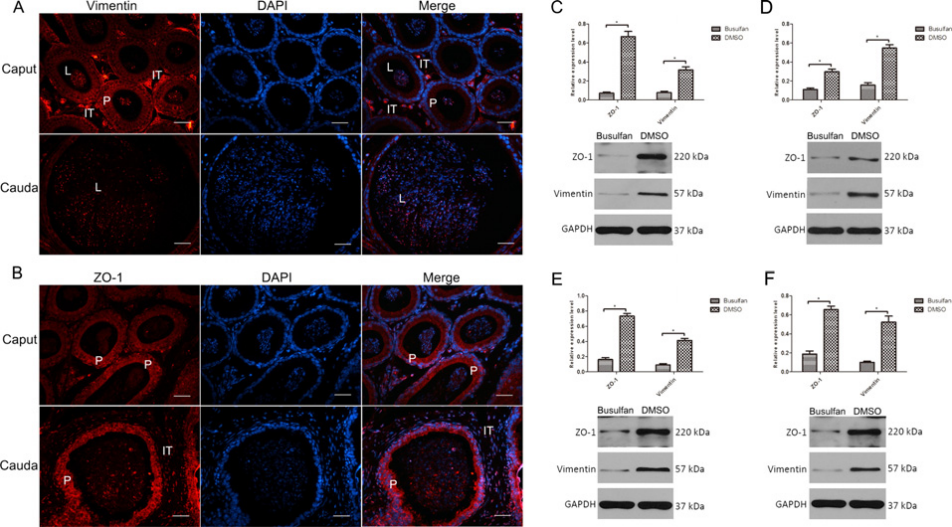
Effect of busulfan treatment on vimentin and ZO-1 protein levels in the mouse epididymis (**A**,**B**) Immunolocalization of vimentin and ZO-1 in the normal mouse epididymis. IT, intertubular space; L, lumen; P, principal cells. Bar: 50 μm. (**C–F**) Protein levels of vimentin and ZO-1 in the busulfan and DMSO groups. The 1st (C), 2nd (D), 3rd (E), and 4th (F) week after treatment. The lower panels show the protein levels in two groups by Western blot. The upper panels show the densitometric evaluation of the independent Western blot. *n*=3, error bars denote ± S.D. *Significant difference (*P*<0.05), busulfan group compared with DMSO group. Bar: 50 μm.

Vimentin was present in the principal cells of the caput epididymidis, with predominant expression in the interstitial portions and in spermatozoa in the lumen of epididymis. Vimentin levels in the principal cells were decreased toward the cauda region of the epididymis, but vimentin remained strongly expressed in the sperm (Figure 3A). ZO-1 was present in the principal cells that form tight junctions with one another both in the caput and cauda epididymidis, and it was also detected in the interstitial part of the cauda epididymidis (Figure 3B).

After a single injection of busulfan, vimentin and ZO-1 protein levels in the whole epididymis declined after 1 week, and were maintained at a low level until the end of the 4th week, similar to the mRNA expression levels detected by qPCR (Figure 3C–F).

### Changes in AR and ER-α expression in the busulfan-treated mouse epididymis

**Figure 4:**
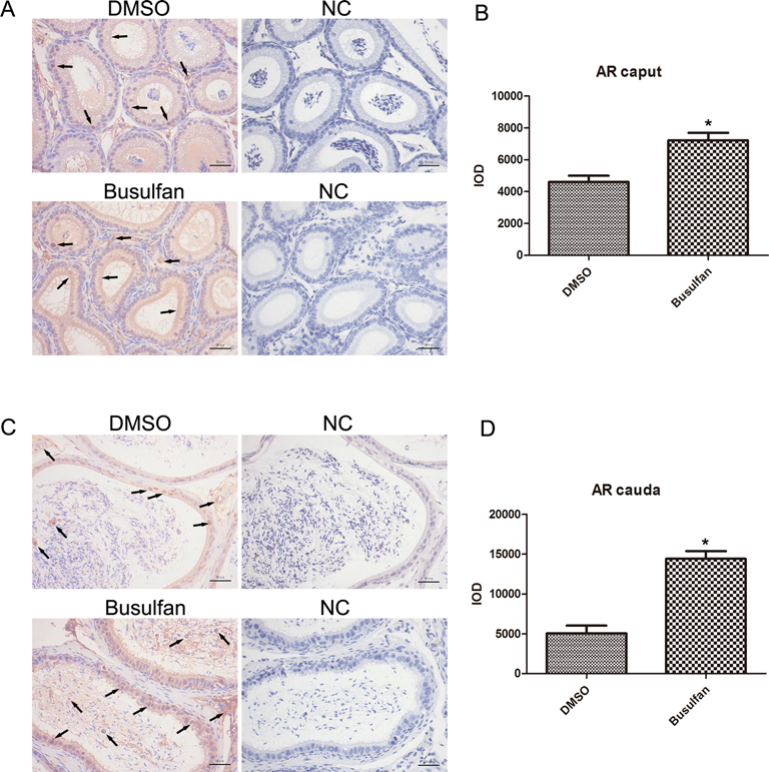
Immunohistochemical staining of AR in the caput and cauda epididymidis at the end of the 4th week (**A**,**B**) AR in the caput epididymidis and quantitative analysis. (**C**,**D**) AR in cauda epididymidis and quantitative analysis. *n*=3, error bars denote ± S.D. *Significant difference (*P*<0.05), busulfan group compared with DMSO group. Arrows: location of AR. NC, negative controls with no primary antibodies. Bar: 50 μm.

**Figure 5:**
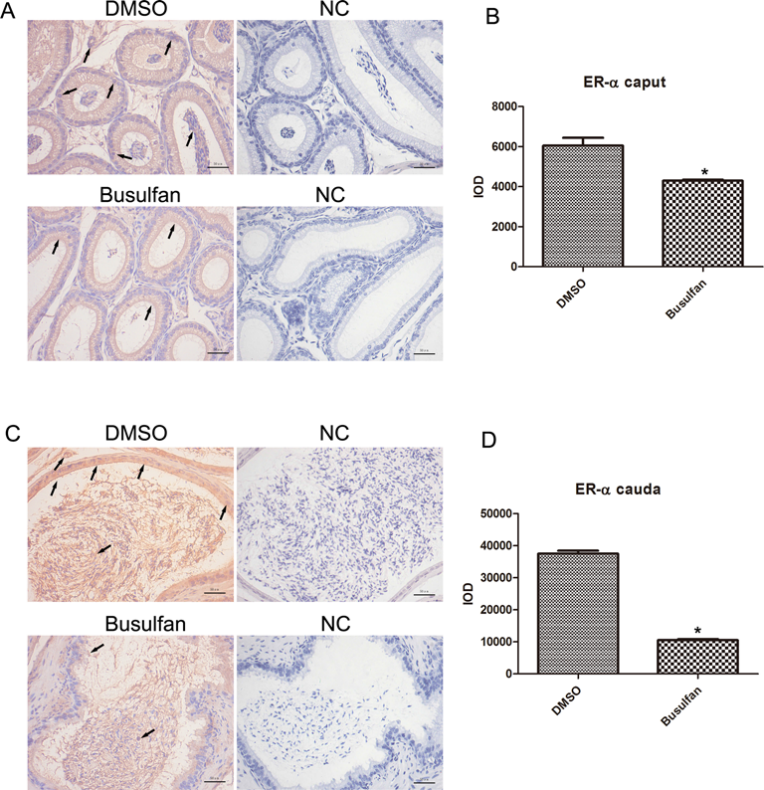
Immunohistochemical staining of ER-α in the caput and cauda epididymidis at the end of the 4th week (**A**,**B**) ER-α in the caput epididymidis and quantitative analysis. (**C**,**D**): ER-α in the cauda epididymidis and quantitative analysis. *n*=3, error bars denote ± S.D. *Significant difference (*P*<0.05), busulfan group compared with DMSO group. Arrows: location of ER-α. NC, negative controls with no primary antibodies. Bar: 50 μm.

Steroid hormones are well-known regulatory factors of epididymal differentiation and function. Therefore, we determined the AR and ER-α expression patterns in the busulfan-treated mouse epididymis. In the caput and cauda regions, AR and ER-α were predominant in the nuclear region of epithelial cells, especially the principal cells in the DMSO groups; but were also detected in interstitial cells and in the cytoplasm of epithelial cells. In addition, spermatozoa in the lumen also showed intense staining for ER-α (Figures 4A,C and 5A,C). After 4 weeks of busulfan treatment, there was a significant increase in total AR expression (Figure 4B,D), whereas the expression of ER-α was markedly reduced (Figure 5B,D), both in the caput and cauda regions. We also observed many round cells positive for AR or ER-α in the lumen of the cauda epididymidis.

## Discussion

As the epididymis is where sperm maturation occurs, it is important to recognize the effects of busulfan treatment on this organ which is an area often overlooked. The present study determined the effect of busulfan treatment on the epididymis. Approximately 4 weeks after busulfan treatment, germ cells in the process of spermatogenesis were cleared from the seminiferous tubules; therefore, we used different time points from week 1 to 4 to observe the effects of busulfan on the epididymis [13].

We found busulfan was damaging the structure and function of the testis and epididymis, which could be the underlying reason for the obvious decrease in testicular and epididymal weights. The toxicity of busulfan to the epididymal epithelium resulted in cellular disorganization and degeneration, the consequent reduction in epithelial height and epididymal tubule diameter, and sloughed apoptotic germ cells in the epididymal lumen in addition to the decreased number of cauda epididymidal sperms. From a toxicological perspective, the epididymis is inherently complicated as its structure and function can be changed both indirectly and directly, as previously reviewed [19].

In the epididymis, epithelial cell–cell interactions are mediated by adhering junctions, gap junctions, and tight junctions, which are necessary for cell adhesion and the formation of the blood–epididymal barrier, respectively [8]. The regulation of these cellular junctions is thought to represent a key determinant in the process of sperm maturation within the epididymis. In particular, the specific intraluminal environment of the epididymis is immunoprotected by the blood–epididymal barrier [20–23]. The toxicity of busulfan to the epididymal epithelium may alter the epithelial cell–cell interactions directly by adversely impacting the expression of junctional proteins. Therefore, we detected the mRNA expression levels of gap junctional proteins (connexion 43) [24–26], tight junctional proteins (occludin and ZO-1) [8,27], and adhering junctional proteins (ICAM, cadherin2, and cadherin3) [8,28], as well as the intermediate filament protein vimentin, which is a component of the cytoskeleton [29]. Generally, we found that the busulfan-treated epididymis exhibited down-regulated expression of ZO-1 and vimentin. ZO-1 is a peripheral membrane protein involved in tight junctions between adjacent principal cells, which are crucial for the formation of the blood–epididymal barrier [30,31]. The decreased expression of ZO-1 indicated an impairment of the blood–epididymal barrier. Vimentin mainly contributes to controlling cell shape changes and cell mechanics [29,32,33]. Previous studies reported that vimentin was associated with the desmosome-like junctions in the testis [34]. Similarly, in the epididymis, the collapse of vimentin caused by busulfan treatment might result in an impaired anchoring function of these junctions between adjacent principal cells, leading to the loss of structural integrity of the epididymal epithelium; however, this speculation requires further confirmation. As shown by immunostaining, vimentin was also located in spermatozoa in the lumen, indicating the decreased expression of vimentin might be related to the reduced number of sperms that enter the epididymis.

However, the notable histological alterations in the epididymis might be secondary to the busulfan toxicity in the testis. It has been extensively reported that testicular lumicrine factors, androgens and estrogens participate in the regulatory process of epididymal differentiation and function [35,36]. Toxicant-induced perturbations in the volume or composition of testicular fluid can also alter cellular interactions in the epididymis by the influence of testicular factors that regulate the expression of the cellular targetting proteins [19].

The physiology of the epididymis relies on testicular and circulating factors that the epididymis is unable to synthesize by itself, such as androgens and estrogens, which mediate their biological effects after interacting with their corresponding nuclear receptors (AR or ERs) and the subsequent recognition of response elements (AREs or EREs) located in the promoters of target genes [4,18,37,38]. AR and ERs (ER-α and ER-β) belong to the nuclear hormone receptor superfamily of ligand-activated transcription factors [36–39]. We found a significant increase in AR expression level 4 weeks after busulfan treatment in the caput and cauda regions of the mouse epididymis. Indeed, androgens are the main modulators of AR expression both in negative and positive directions based on many cell lines analyzed [40–43]. Leydig cells are the source of androgen production by the testis, and we observed that the morphology of Leydig cells was influenced after busulfan treatment. The dysfunction of Leydig cells could affect testicular androgen production, which might induce the expression of AR in the epididymis as a compensatory mechanism. However, previous studies have reported that testosterone levels remain constant throughout busulfan treatment and that it does not alter the steroidogenic environment in mice [13,44,45]. The exact molecular mechanisms underlying the regulation of AR expression in the epididymis is still poorly understood. Epididymal gene expression is segmentally regulated by androgens, and these genes encode proteins involved in epididymal functions [4,46,47]. After the disruption of the epididymal morphological structure and blood–epididymal barrier caused by busulfan treatment, the increased expression of AR in the epididymis may be an androgen-mediated compensation mechanism to modulate gene expression and activate signal pathways related to epididymal functions. Furthermore, the secretion of ABP could be influenced by impairment of spermatogenesis caused by busulfan [48], which might be related to the changed AR expression pattern.

Much evidence suggests that the epididymis is also an important target for estrogen [36,37], which might play a role in the regulation of adherens and tight junctions to sustain the integrity of the epididymal epithelium [49]. In contrast with AR, we observed that treatment with busulfan decreased ER-α expression both in the caput and cauda epididymidis. The level of estrogen in epididymis may regulate the expression of its receptors [50]. The sources of estrogen in the epididymal lumen are spermatozoa and the epididymal epithelium, which both exhibit aromatase activity that generates estrogen from androgen [37,51–55]. As an important lumicrine factor, spermatozoa can cross-talk with the epididymal epithelium to regulate epididymal physiology and protein secretion [56,57]. Busulfan treatment can alter the quantity and quality of the spermatozoa that enter the epididymis by its toxic effects on spermatogenesis. Therefore, we postulate that the biosynthesis of estrogen is influenced by busulfan, resulting in decreased ER-α level. The round cells that were stained positive for AR or ER-α in the lumen of the cauda epididymidis might be detached Sertoli cells and germ cells from the testis. In summary, the balance of androgen and estrogen receptors is necessary for normal epididymal epithelium function, and this was altered after busulfan treatment, which might be secondary to the testicular lesion.

## Conclusion

Our study showed that the toxicity of busulfan is not restricted to differentiated spermatogonia in the testes but also affects the epididymis, as demonstrated by disruption of the epididymal epithelial structure, decreased vimentin and ZO-1 levels, and opposite changes of AR and ER-α expression after busulfan treatment. Although the toxic effects of busulfan on the epididymis might be secondary to the testicular lesion, we cannot say whether direct pathological effects occur in the epididymis without further studies. Therefore, future studies should ascertain the exact mechanisms by which busulfan causes all these effects on the epididymis and whether they are direct or indirect.

## Supporting information

**Supplemental Figure 1:** Histological examination of seminiferous tubules in mice following busulfan and control treatment. The 1^st^ (A), 2^nd^ (B), 3^rd^ (C) and 4^th^ (D) week after treatment. Bar: 50 μm.

## Abbreviations

ABP: androgen-binding protein
AR: androgen receptor
ER-α: estrogen receptor-α
IOD: integral optical density
TUNEL: terminal deoxynucleotidyl transferase dUTP nick-end labeling
ZO-1: zonula occludens-1
