# Supplementary material for: Busulfan administration produces toxic effects on epididymal morphology and inhibits the expression of ZO-1 and vimentin in the mouse epididymis
Source: Biosci Rep. 2017 Dec 12;37(6):BSR20171059. doi: 10.1042/BSR20171059 (PMC5725615; doi:10.1042/BSR20171059)
Supplement: Supplementary file 1 [file bsr20171059_Supp1.pdf]

Figures

Supplemental Figure 1. Histological examination of seminiferous tubules in mice following busulfan and control treatment. The 1st (A), 2nd (B), 3rd (C) and 4th (D) week after treatment. Bar: 50 μm.

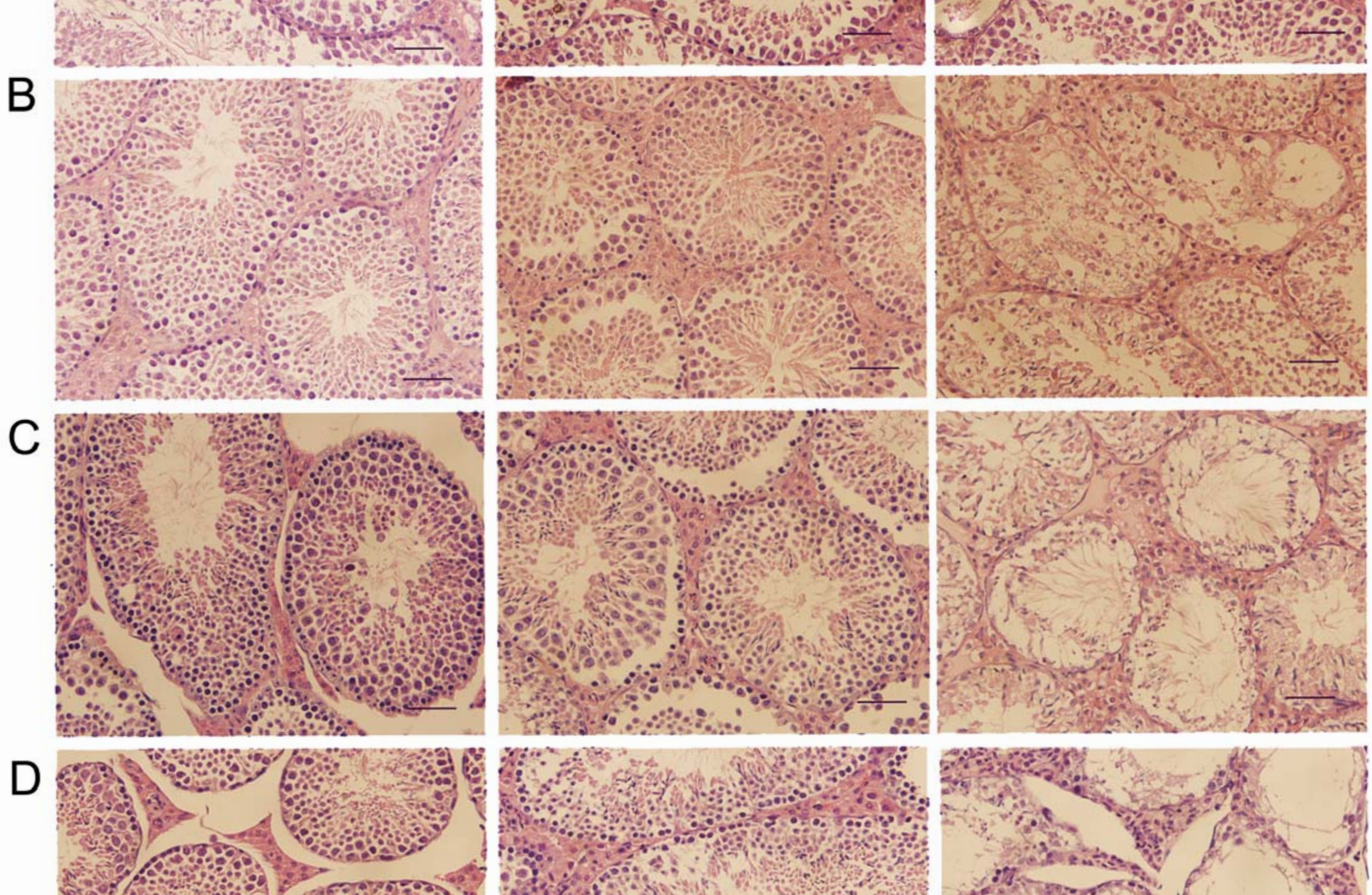

Saline
DMSO
Busulfan
A
B
C
D
